# Prognostic Value of Association of Copy Number Alterations and Cell-Surface Expression Markers in Newly Diagnosed Multiple Myeloma Patients

**DOI:** 10.3390/ijms23147530

**Published:** 2022-07-07

**Authors:** Mihaiela L. Dragoș, Iuliu C. Ivanov, Mihaela Mențel, Irina C. Văcărean-Trandafir, Adriana Sireteanu, Amalia A. Titianu, Angela S. Dăscălescu, Alexandru B. Stache, Daniela Jitaru, Dragoș L. Gorgan

**Affiliations:** 1 Biology Department, Faculty of Biology, “Alexandru Ioan Cuza” University of Iasi, 700506 Iasi, Romania; dragosloredana.z@gmail.com (M.L.D.); stache.bogdan@gmail.com (A.B.S.); 2 Molecular Diagnosis Department, Regional Institute of Oncology, 700483 Iasi, Romania; iuliuic@gmail.com (I.C.I.); adryas@gmail.com (A.S.); 3 Center for Fundamental Research and Experimental Development in Translation Medicine—TRANSCEND, Regional Institute of Oncology, 700483 Iasi, Romania; mentelmihaela@gmail.com (M.M.); trandafirina.bi@gmail.com (I.C.V.-T.); 4 Immunophenotyping Department, Regional Institute of Oncology, 700483 Iasi, Romania; 5 Department of Hematology, Regional Institute of Oncology, 700483 Iasi, Romania; merticariuamaliaandrea@yahoo.ro (A.A.T.); angdascalescu@yahoo.com (A.S.D.); 6 Department of Hematology, “Grigore T. Popa” University of Medicine and Pharmacy, 16 University Street, 700115 Iasi, Romania

**Keywords:** multiple myeloma, plasma cells, MLPA, flow cytometry

## Abstract

Multiple myeloma results from the clonal proliferation of abnormal plasma cells (PCs) in the bone marrow (BM). In this study, the cell surface expression markers (CD) on atypical PCs (detected by multiparametric flow cytometry (MFC)) were correlated with copy number alterations (CNAs) in the genome (detected by multiplex ligation-dependent probe amplification (MLPA)) to assess their impact on prognosis in newly diagnosed MM patients. Statistically significant results were obtained when different stages of PC maturation (classified based on CD19 and CD81 expression) were associated with CD117 expression and identified CNAs. In the intermediately differentiated PC group (CD19(−) CD81(+)), patients who didn’t express CD117 had a lower median progression free survival (PFS) (*p* = 0.024). Moreover, within this group, patients with less than three adverse CNAs, which harbor CD117, had a better outcome with a PFS of more than 48 months compared with 19 months (*p* = 0.008). Considering all the results, our study suggested the need to integrate both the CD markers and copy number alterations to evaluate the prognosis of newly diagnosed multiple myeloma patients.

## 1. Introduction

Multiple myeloma (MM) represents around 10% of all hematological malignancies [1,2] and results from the clonal proliferation of abnormal plasma cells (PCs) in the bone marrow (BM) [3]. MM occurs mainly in the elderly population (median age at diagnosis: 70 years) [4] and is characterized by anemia, myelosuppression and bone destruction, as well as clinical consequences from the paraproteinemia on kidney function and other organ systems [5].

MM is always preceded by a condition known as monoclonal gammopathy of undetermined significance (MGUS) that displays a lifelong rate of progression of 1% per year [6,7] and is characterized by less than 10% PCs BM infiltration [8]. Smoldering multiple myeloma is the second stage in the development of the disease, where the infiltration of BM with clonal PCs increases compared with MGUS, but terminal organ damage symptoms related to myeloma are not yet present [9]. The genome of MM is complex and heterogeneous, with a high frequency of structural variants and copy number alterations (CNAs) [10]. The CNAs are one of the most recurrent genomic events in this pathology and include, in the case of hyperdiploid (HRD) MM, trisomy of certain chromosomes with odd numbers 3, 5, 7, 9, 11, 15, 19 and 21, whereas other non-HRD MM patients usually show chromosomal rearrangements, especially involving the immunoglobulin heavy chain (IgH) locus. The hemizygous deletion of chromosome (chr) 13 is the most common deletion in both HRD and non-HRD MM patients [11,12]. Cell surface antigens used to characterize abnormal PCs by flow cytometry are CD38, CD138, CD19, CD56, CD117 and CD45. Of these, the most specific marker for myeloma cells is CD138 (Syndecan-1). CD138 is a transmembrane proteoglycan that is expressed on the basolateral surface of endothelial cells, embryonic mesenchymal cells, vascular smooth muscle cells, endothelium, neuronal cells and pre-B cells. Its expression is lost during differentiation, yet CD138 is re-expressed in the plasma cell stage. This antigen is used for sorting atypical PCs plasma cells to enrich samples from MM patients [13].

CD19 is a B-lymphocyte-specific antigen and atypical plasma cells are predominantly negative for this marker [14,15]. It is a transmembrane glycoprotein from the immunoglobulin family and is encoded by a gene located at position 16p11.2 [16]. In MM, the absence of CD19 expression was correlated with the low expression of the PAX5 gene [17]. The presence of CD19 (rare) in this pathology was associated with the presence of small plasmocytes with translocation t(11:14) [18].

CD81 is a tetraspanin protein that regulates CD19 expression [19]. The gene responsible for this protein is located at position 11p15.5 [20]. The expression of CD81 in atypical plasmocytes varies depending on the stage of cell maturation [21] and was correlated with a negative prognosis in SMM and MM [22,23].

CD117 is a tyrosine kinase receptor encoded by a proto-oncogene, c-kit, located at position 4q12 [24]. It was found to be expressed on most myeloid lineage precursors, a part of T-lineage ALL and on some atypical plasmocytes [25,26]. The CD117 expression in MM patients was associated with a better prognosis [27].

In this study, the PCs from newly diagnosed MM patients were analyzed using multiplex ligation-dependent probe amplification (MLPA) and multiparametric flow cytometry (MFC) methods. Genomic studies so far [28,29,30] in MM have generally highlighted the effect of structural chromosomal abnormalities, but they were taken only as an independent prognostic factor. Our aim was to correlate both the expression of PCs specific markers and CNAs with progression free survival (PFS) and overall survival (OS), to identify new criteria for risk stratification in MM patients.

## 2. Results

### 2.1. CNAs Identified

Initially, the study included 389 patients who were referred for assessment of plasma cell clonality by flow cytometry. All patients were analyzed on the basis of clinical data, biochemical and hematological parameters and by applying established exclusion criteria (see methods). The characteristics of the study group are listed in Table 1. Out of 156 patients with confirmed MM, only 107 were investigated by both flow cytometric analysis and MLPA.

The MLPA kit used allowed the identification of both known adverse CNAs and HRD, which has been reported in the literature to have a positive prognosis [31,32,33].

In the studied group the most frequent genetic abnormality was HRD, identified in 49.5% cases, followed by dup1q (40.1%). The del13q was the most frequent chr loss in 38.3% patients, whereas 27.1% of them did not show any genetic modification among the targeted sequences (Table 2).

**Figure 1:**
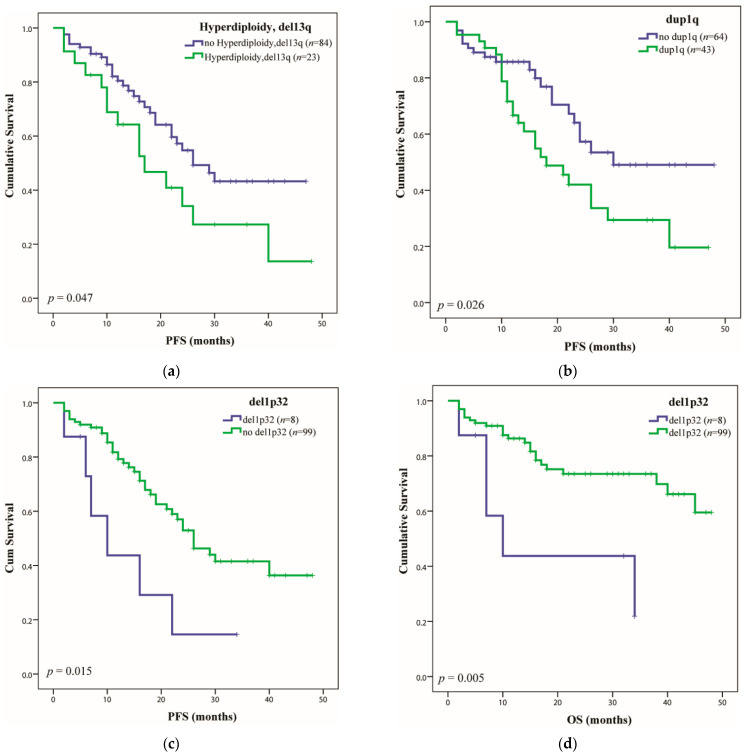

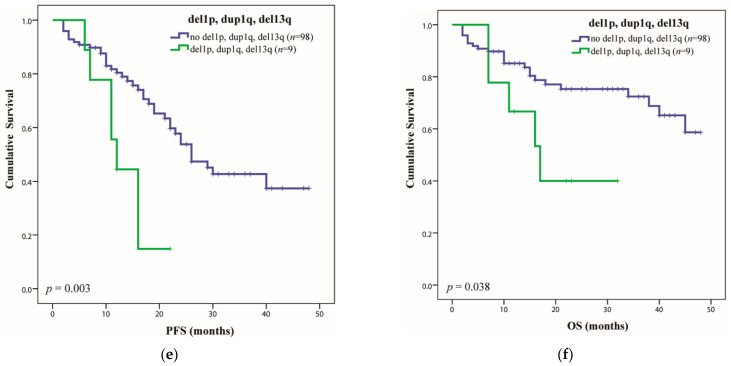
Overall survival (OS) and progression-free survival (PFS) of multiple myeloma (MM) patients correlated with the identified CNAs. The Kaplan–Meier estimates of (**a**) PFS with the presence or absence of HRD with del13q; (**b**) PFS for dup1q positive group; (**c**) PFS and (**d**) OS of the group of patients that presented del1p32; (**e**) PFS for del1pdup1qdel13q positive group; (**f**) OS for del1pdup1qdel13q positive group.

Regarding HRD (duplications of probes in regions of chromosomes 5q, 9, 15), the highest frequency of duplications was dup9 (*n* = 42), followed by dup15 (*n* = 40) and dup5q (*n* = 34). HRD was associated with del13q in 43.39% (*n* = 23/53) of cases and determined a shorter median PFS of 17 months (*p* = 0.047), as previously observed by Shah et al., 2018 [34], (Figure 1a). Identification of 1q gain, as an independent factor, was associated with a poor prognosis [35], with a median PFS of 18 months (*p* = 0.026) (Figure 1b).

Concerning the second most common chr loss, namely del1p (*n* = 23, patients with at least two probes from nine deleted in this region), the region 1p21 was the most frequently affected, in 91.3% of cases (*n* = 21/23), while whole (or almost whole) arm deletion was present in only 34.7% of them (*n* = 8/23). The poorest prognosis in this group was conferred by del1p32 (7.5% cases), as mentioned by Hebraud et al., 2014 [36], with a median PFS of 10 months (*p* = 0.015) (Figure 1c) and median OS of 10 months (*p* = 0.005) (Figure 1d). A group of del1p patients also had HRD (56.5%) and/or del13q (47.8%). Association between del1p and dup1q was found in 12 samples (27.9% of dup1q positive group), 9 of which also presenting del13q and having a poor prognosis, with a median PFS of 12 months (*p* = 0.003) and median OS of 17 months (*p* = 0038) [37] (Figure 1e,f).

The association between del17p and del13q was found in two cases, showing a negative impact on the median PFS (6 months) (*p* = 0.005) and for OS (7 months) (*p* = 0.011) [38].

**Figure A1:**
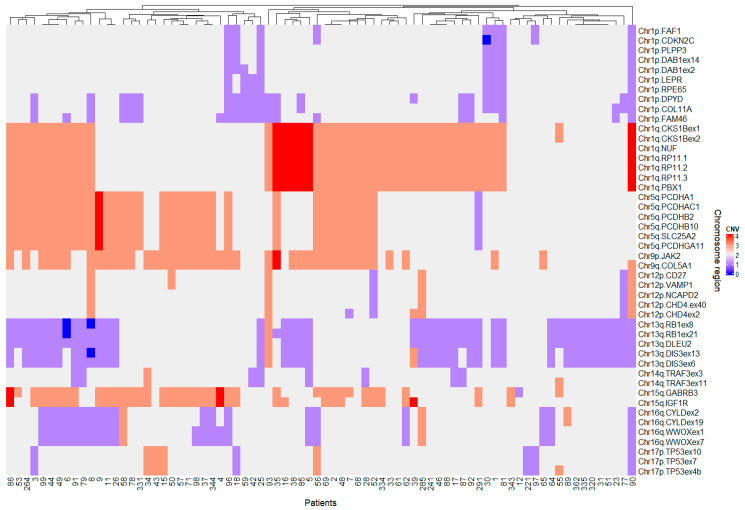
Heatmap of the CNA distribution (the patients who didn’t present any CNAs were excluded). 0-homozygous deletion, 1-heterozygous deletion, 2-normal, 3,4-duplications.

**Figure A2:**
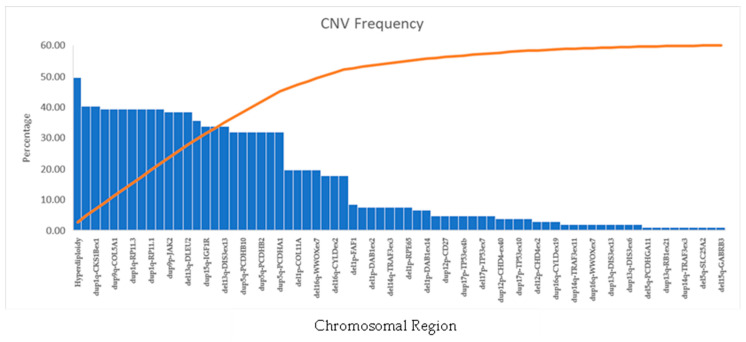
Frequency of the CNAs in the study group.

**Figure A3:**
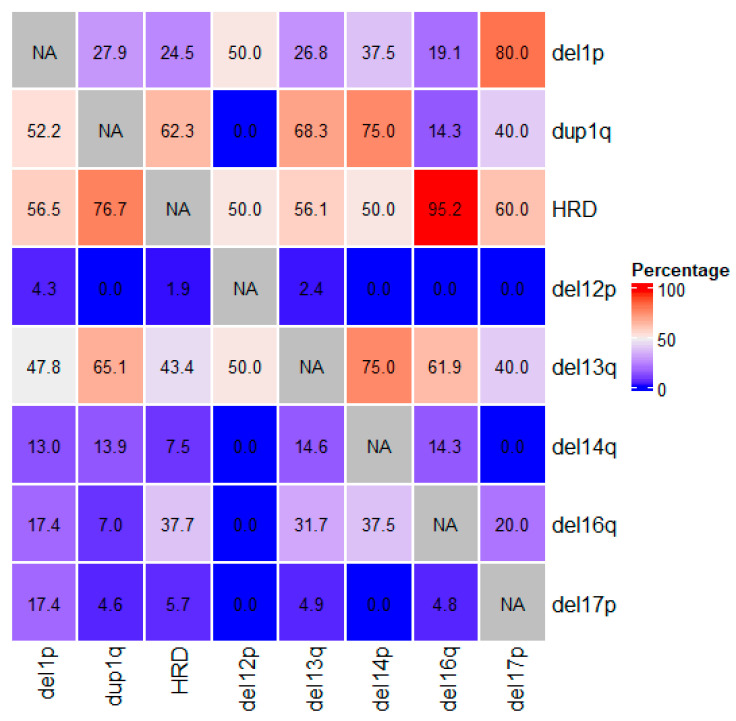
Heatmap of pairwise association between CNAs. The percentage was calculated from the positive group. Each row is a group.

**Figure A4:**
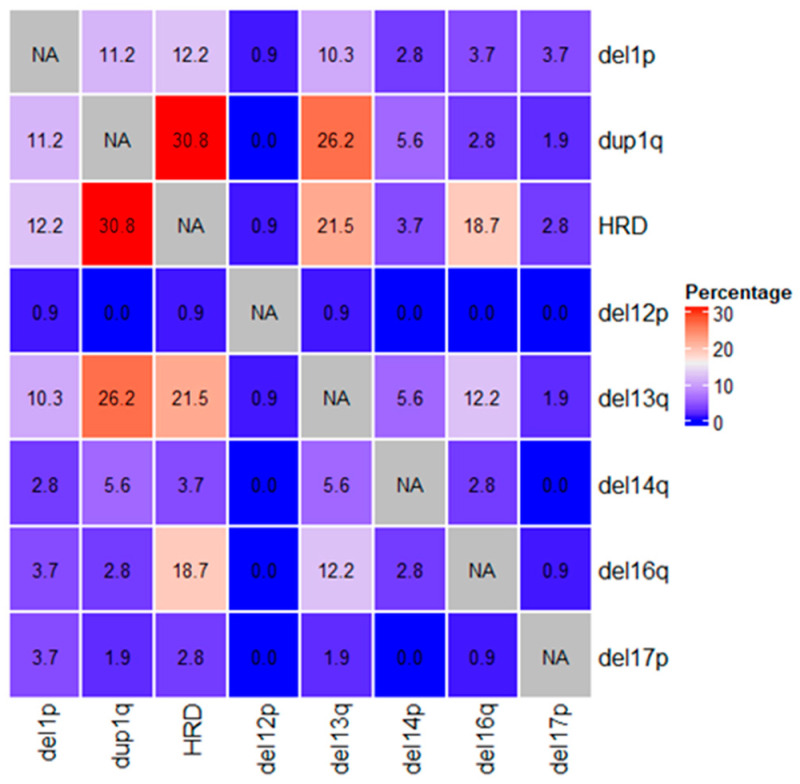
Heatmap of pairwise association between CNAs. The percentage was calculated from the whole group.

The distribution, frequency and pairwise association prevalence between CNAs and their frequencies are represented in Appendix A (Figure A1, Figure A2, Figure A3 and Figure A4).

**Figure 2:**
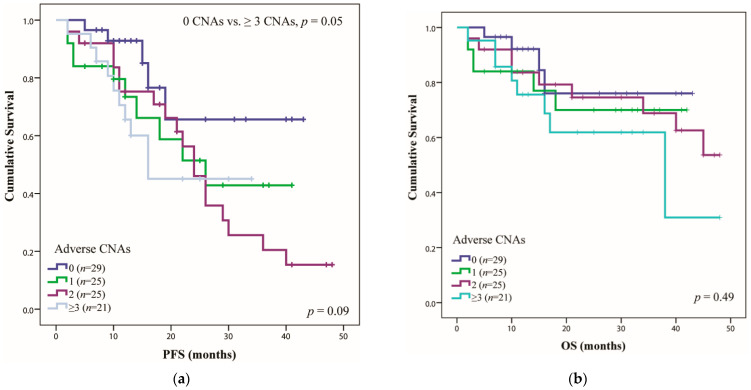
The progression-free survival (PFS) (**a**) and overall survival (OS) (**b**) correlated with adverse CNAs. The median PFS and OS were calculated using Kaplan–Meier analysis for survival time. Where, 0—no adverse DNA damage, 1—one adverse CNA, 2—two adverse CNAs and ≥3—at least three adverse CNAs.

In terms of the CNA accumulation impact, it seems that the association of at least three adverse alterations had the greatest impact on PFS, with a median duration of 16 months (*n* = 21), compared with patients without adverse CNAs (PFS greater than 48 months) (*p* = 0.05) (Figure 2a). The OS was lower only in the case of patients with at least three adverse DNA alterations (38 months), whereas the rest of the patients exceeded the study timeline (Figure 2b). In the group without adverse CNAs (*n* = 29), only patients with no genetic alterations were included (thus seven patients who had only HRD without other adverse CNAs were excluded). The presented results are consistent with the data presented previously by other studies [39,40,41].

### 2.2. Correlation between Aberrant PC Immunophenotype and CNAs

**Figure A5:**
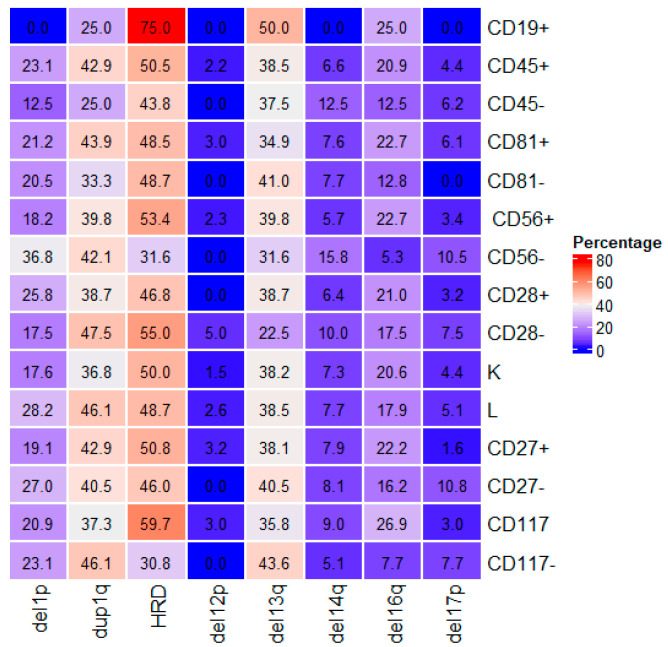
Pairwise association between CNV and CD.

The monoclonal PCs were identified and phenotypically characterized by MFC. The frequency of the CD expression and the number of samples investigated for each marker are listed in Appendix B (Table A1 and Figure A5). In our group, CD19 was predominantly negative, except for four cases. Among the cell surface markers analyzed, the expression of the following markers, as prognostic indicators, has been considered: CD81, CD117 and CD19 correlated with the identified CNAs.

The CD81 marker was expressed in 62.8% of patients, being an adverse marker [22] with median OS of 45 months (*p* = 0.012). More than 30% of patients had either del13q or dup1q. Association with the latter had a significantly negative impact on median OS (38 months) (*p* = 0.002) and PFS (14 months) (*p* = 0.001). In patients where CD81(+) was associated with more than three adverse CNAs (*n* = 13), the PFS was decreased to a median of 13 months (*p* = 0.045) and an OS of 38 months (*p* = 0.017) (Table 3).

**Figure 3:**
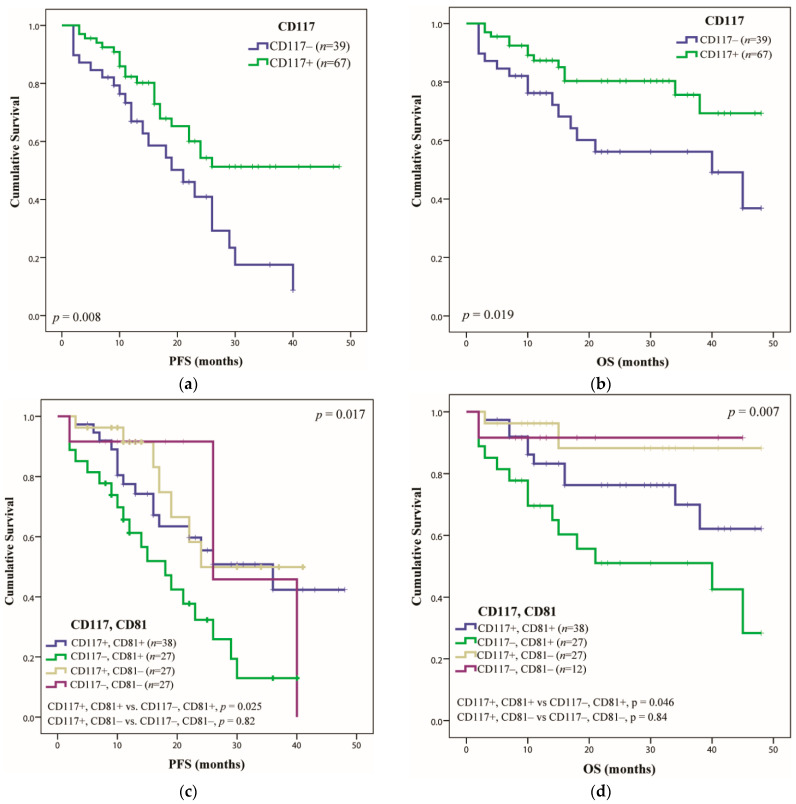

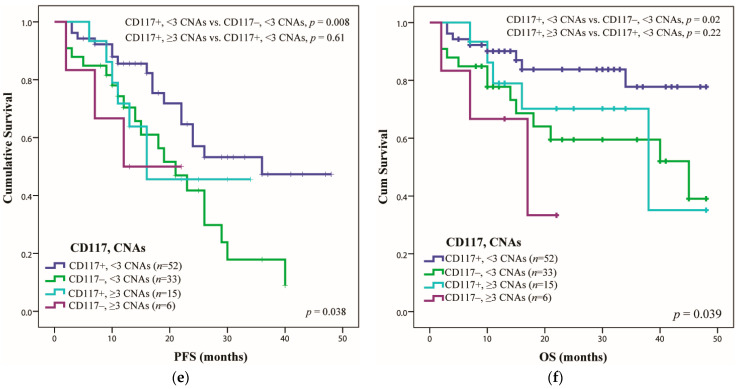
Multiple myeloma patients’ survival rate, taking into account the expression of CD117 marker, by Kaplan–Meier estimates: (**a**) PFS and (**b**) OS of MM patients that expressed CD117 compared with patients who do not express this marker; (**c**) PFS and (**d**) OS for patients who expressed CD117 correlated with the presence or absence of the CD81 marker; (**e**) PFS and (**f**) OS for the correlation of CD117 with adverse CNAs.

The expression of the CD117 marker was documented as a positive prognostic factor [22], and this was also observed by our group. Thus, it was identified in 67 patients and had a favorable impact for PFS (more than 48 months, *p* = 0.008) and OS (more than 48 months, *p* = 0.019) (Figure 3a,b). CD117 expression also improved the outcome of patients who expressed CD81(+), thus, the median PFS increased from 18 months to 36 months in cases of CD117(+) CD81(+) vs. CD117(−) CD81(+) (*p* = 0.025, HR 0.48 (95% CI 0.23–0.90)) and the median OS from 40 months to more than 48 months (*p* = 0.046, HR 0.44 (95% CI 0.19–0.99). There was no difference in the PFS or OS within the CD81(−) group with or without CD117 expression (*p* = 0.82/*p* = 0.84) (Figure 3c,d).

Next, an association analysis was performed for CD117 with at least three adverse CNAs. CD117 expression had a positive impact in the group of fewer than three CNAs. Thus, in this group, the CD117 expression increased the PFS from 21 to 36 months (*p* = 0.008) and OS from 45 to over 48 months (*p* = 0.02) (Figure 3e,f). In the group with more than three CNAs, the expression of CD117 showed no statistical significance, maybe due to the low number of patients in this group.

**Figure A6:**
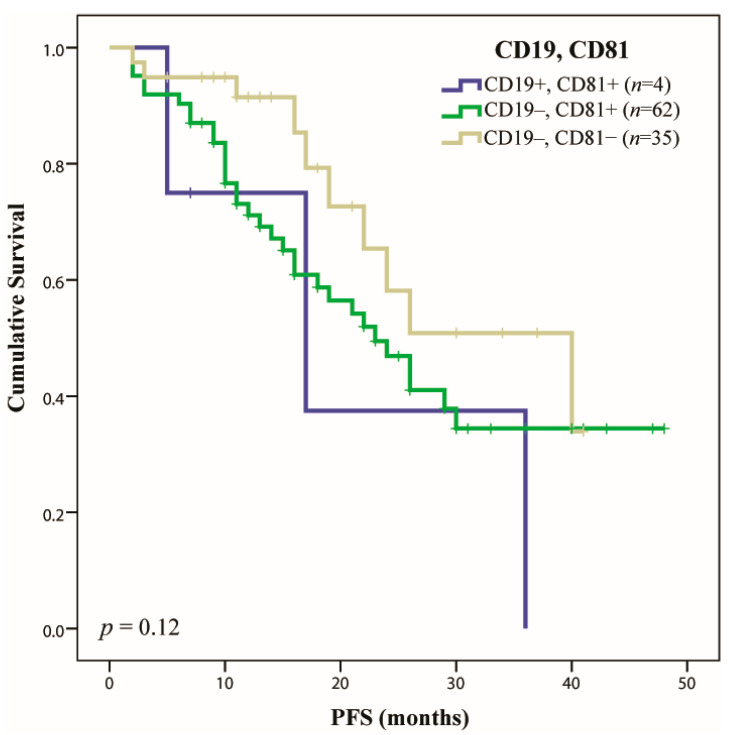
Multiple myeloma patients’ survival, considering the co-expression of CD19 and CD81 markers by Kaplan–Meier analysis.

**Figure A7:**
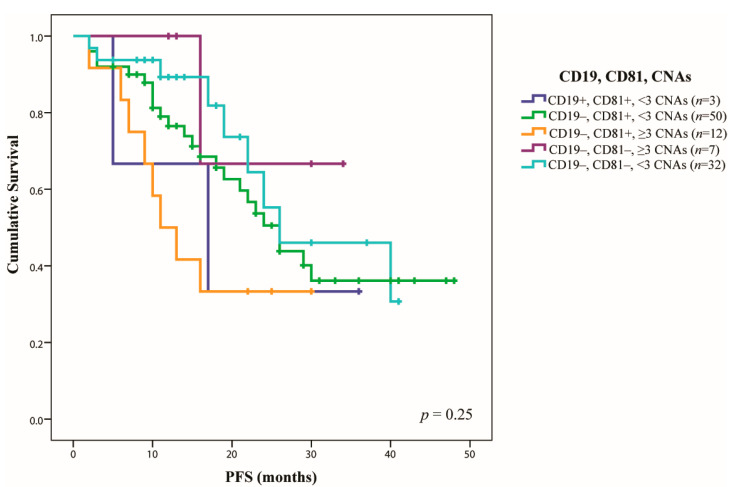
Survival by Kaplan–Meier analysis of association between CD19–CD81 and at least three adverse DNA damages (PFS). From the analysis we excluded the group CD19(+), CD81(+), ≥3 adverse CNAs, being represented be only one patient.

The maturation stages [42] of atypical PCs (CD19(−) CD81(+)—defined intermediate; CD19(−) CD81(−)—defined differentiated and CD19(+) CD81(+)—defined little or non-differentiated) were analyzed. The groups were compared with each other, then with either CD117 or adverse CNAs, or all together. The pairwise association with CNAs is listed in Table 4. Within the PC groups or when they were investigated with adverse CNAs no statistical information was obtained (Appendix B, Figure A6 and Figure A7). This could be due to other factors (like translocations or point mutations) or to the low number of patients.

**Figure 4:**
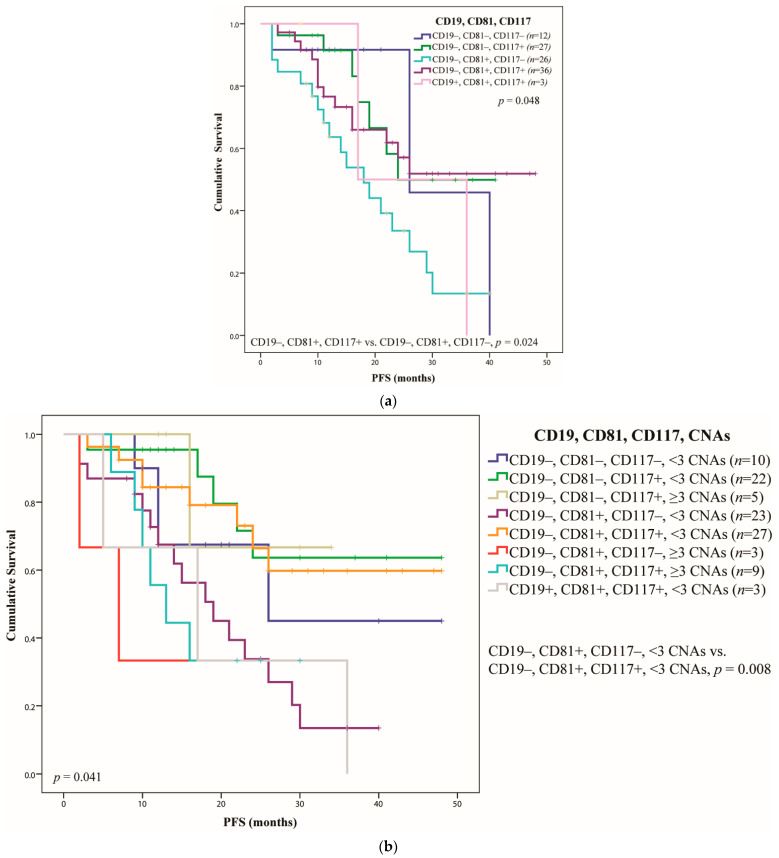
Correlation of PC maturation stages with CD117 (**a**) and both the expression of CD117 and adverse CNAs (**b**). The PFS was estimated by Kaplan–Meier analysis.

In Figure 4a it can be observed that when different PC maturation stages [42] were compared with CD117 expression, the intermediately differentiated group that harbor this marker had a better survival, with a PFS of more than 48 months versus only 18 months for the CD117(−) group (*p* = 0.024).

Next, the PCs were divided according to the expression of CD19 and CD81 markers, and then correlated with both adverse CNAs and CD117 (Figure 4 and Table 5). Thus, it was observed that in the group of intermediately differentiated PCs (CD19(−), CD81(+)) the expression of CD117 had a favorable outcome when less than three adverse CNAs were identified. Thus, CD117 expression increased the PFS from 19 months to more than 48 months (*p* = 0.008). When more than three CNAs were present, CD117 expression had no statistical influence over the PFS and/or OS, possibly due to the low number of patients in this group, suggesting the need for larger cohort studies.

The overall *p* = 0.045 of the compared subgroups (Figure 4) suggested an increase in outcome when CD117 was expressed, regardless of the adverse CNAs, more specifically for intermediately differentiated group. At the same time, our study suggests the need to integrate both the CD markers and copy number alterations in order to evaluate the prognosis of newly diagnosed multiple myeloma patients.

## 3. Discussion

Genomic instability, involving complex numerical and structural abnormalities, is a feature of atypical PCs within MM [43,44]. FISH, karyotypes and cytology are known to be the gold standard for the diagnosis of MM [45]. A better understanding of additional techniques will lead to a more accurate diagnosis and personalized treatment [33,43]. Thus, we set out to investigate whether techniques such as MLPA associated with flow cytometry immunophenotyping can provide valuable additional information.

According to Xiaofei et al. 2021 [46], there is a remarkable correlation of data between iFISH and MLPA, with an overall consistency of 97.1% (1354 results out of 1395 comparisons were concordant). However, there were some discrepancies in resolution, point mutations and subclones caused by distinct probes employed in both methods. Furthermore, it is well known that iFISH analysis is only able to detect deletion or amplification of sequences larger than 20–50 kb [47], whereas MLPA can recognize sequences of 50–100 nt in length, enabling its application for DNAs even in case of higher template fragmentation [48]. Thus, the need to use alternative methods arises, both in multiple myeloma [49,50] and in other hematological diseases.

MFC has the advantage to both differentiate between normal and clonal PCs from the BM plasma cell compartment and identify aberrantly expressed markers in patient samples [2,13].

In our study, the PCs were first selected as double positive for the CD38 and CD138 markers and then the monoclonality was evaluated by light chain restriction. Furthermore, monoclonal PCs were phenotypically characterized both in terms of under-expressed and over-expressed markers (CD19, CD27 and CD81; and CD56, CD28 and CD117, respectively). Next, the CNAs in the plasmocytes of newly diagnosed MM patients were identified by the MLPA technique. The obtained results were then compared to establish different prognostic patterns.

CNAs of chromosome 1 had the most significant impact as independent prognostic factors in the targeted group, according to the results reported in the literature [51,52,53]. Del1p confers an adverse prognosis, especially if the 1p32 region (DAB1 gene) was involved. The incidence of these abnormalities was lower than in other studies, 21.4% versus 30% [54,55]. The loss of 1p12, which is reported as an adverse prognostic factor in myeloma [56], with an incidence of ~13% of MM patients [57], had a higher incidence in our group (17.76%, n = 19), but with no statistical significance for OS and PFS.

In 40.1% of patients a 1q gain was identified, which conferred an adverse prognosis as previously reported in the literature [58,59]. This gain is often associated with 1p deletion, having a negative prognosis and a short OS [58].

Hyperdiploidy (HRD) is one of the basic criteria in classifying chromosomal abnormalities in MM, with an incidence of about 50%, and confers a good prognosis [60,61]. In our study, HRD had a similar frequency of 49.53% [62].

A 13q deletion worsens the prognosis in MM [63], being the most frequent chr loss in our study and it made a difference when it was associated with del1p, dup1q or HRD.

Although del17p, as a bad prognosis indicator, has been reported with an incidence of 8% [62], in the studied group it had an incidence of only 4.67% and was correlated with a shorter PFS and OS prognosis (7 months).

Previous studies showed that chromosomal abnormalities, in different proportions and combinations, can affect the prognosis of MM patients [59], a fact that was also observed in the studied patient group, where the association of del1p with dup1q and/or del13q resulted in an outcome poorer than each alteration taken individually.

Shah et al. [34] divided MM by the presence of cumulative adverse DNA lesions into three groups: with zero adverse lesions, with one adverse lesion and with two adverse lesions. This concept of cumulative lesions, used also by other researchers [64], made the diversity of cytogenetical abnormalities more reliable for clinical practice. In the same way, taking only the adverse CNAs, the present study group was divided into four subgroups (no adverse CNAs, one adverse CNA, two adverse CNAs and at least three adverse CNAs). The better PFS and OS was found in the no adverse CNAs group as previously mentioned, with a median survival of over 48 months [56]. The one CNA and two CNA groups had similar outcomes. The worst survival was observed when at least three CNAs were present, with a PFS of 16 months and an OS of 38 months.

Regarding cell surface markers, it is well known that CD81 indicates a poor prognosis in myeloma patients [42,65], whereas CD117 is correlated with a positive prognosis [66]. However, their co-expression was linked to different survival outcomes [22], which was also valid for our study too, in which the worst prognosis was related to CD117(−) CD81(+). The association of CD81(+) with the studied adverse CNAs reduced the OS from 48 months to 34 months in the case of association with del1p and to 38 months in the case of more than three adverse CNAs. When three adverse CNAs also showed CD81(+), survival duration decreased from 16 months to 13 months, enhancing the negative prognostic factor of CD81 expression.

CD117 (c-kit) is a tyrosine kinase receptor that was found to be expressed on PC in approximatively 30% of MM [22]. Usually, it was associated with good prognosis, HRD, and lack of chromosome 14 translocations [67,68]. Given that CD117 is a favorable prognostic factor, its role has been analyzed with CD81, adverse CNAs and with different stages of PC maturation. Thus, its expression had a positive impact in the CD81(+) cohort and when less than three adverse CNAs were identified.

Regarding the PC maturation stages, our cohort was divided into three groups depending on the CD19 and CD81 co-expression, similarly to Paiva et al. [42]: CD19(−) CD81(−) defined as differentiated stage; CD19(−) CD81(+) as intermediate stage; and CD19(+) CD81(+) as little or non-differentiated stage. The majority of studied patients (59.04%) had atypical PCs at the intermediate stage (CD19(−) CD81(+)).

The CD19 antigen of the B cell lineage is usually not expressed on malignant PCs but found on normal PCs [43]. Yet, in some rare cases, the CD19 antigen was found in atypical PCs, and it is known to correspond to a pre-malignant phase [69]. When PC groups were compared either with each other or with adverse CNAs, there was no statistical difference in PFS or OS, possibly due to the low number of patients in some groups or because of other factors not assessed in our study (e.g., translocations, point mutations, etc.).

When we analyzed the PC groups with both adverse lesions and CD117 expression, relevant results were obtained only for the CD19(−) CD81(+) group. Thus, CD117 expression had a positive prognostic value when less than three adverse CNAs were identified.

Overall, CD117 expression improved the outcome in the intermediately differentiated PCs, regardless of the number of DNA alterations. As a result, the favorable prognostic factor CD117 should be taken into consideration for the staging algorithm and disease follow-up. Consequently, this study shows that CD19 and CD81, which assign MM patients to three distinct groups of PCs differentiation, further associated with cumulative CNAs and CD117, allows the identification of new important prognostic subgroups, which may represent additional targets for specific studies and even for specific therapies.

## 4. Materials and Methods

### 4.1. Study Design and Classification Criteria

The study was performed at the Regional Institute of Oncology (IRO) Iasi, Romania between 2018 and 2021 (48 months). Ethical approval for this study was obtained from the local ethics commission (no: 6/16.01.2018) and the written consent to participate in studies involving human participants was signed by each patient. All candidate samples (bone marrow (BM) aspirates from patients with suspected MM) were first submitted for microscopic examinations to indicate the presence of atypical PCs. All BM aspirates were collected on EDTA anticoagulant and analyzed for diagnosis before administration of any treatment. The three criteria applied for selection were: (i) newly diagnosed with active MM; (ii) PC infiltration into BM higher than 10%, evaluated by cytology; and (iii) the PCs evaluated by flow cytometry higher than 1.5%. At the same time, all patients who died before the implementation of treatment were excluded from the study.

### 4.2. Flow Cytometry Assessment

The PCs immunophenotype was assessed using markers suggested by the EuroFlow Consortium for plasma cell disorders (CD38(FITC), CD138(PO), CD45(PB), CD19 (PECy7), CD27(PerCP-Cy5.5), CD28(PE), CD56(PE), CD81(APCH7), CD117 (APC), cyIgL (APCC750) and cyIgK (APC)) [70]. All samples were stained following the intracellular and surface staining EuroFlow sample preparation protocols [71,72,73,74] within 24 h from collection. Briefly, 50 µL of sample per tube (2 tubes) were stained with surface markers for 15 min at room temperature (RT), in the dark. After washing with 1X PBS supplemented with 0.5% bovine serum albumin (BSA) and centrifugation at 540× *g* for 5 min, samples were successively treated with Reagent A and B of FIX & PERM^TM^ solution, for 15 min each, followed by washing steps. Next, the intracellular markers were added for 15 min at RT, in the dark. Then, the cellular pellet, obtained after washing and centrifugation, was resuspended in 250 µL of BD FACS Flow (BD Bioscience, Erebodegem, Belgium). An average of 100,000 cells were acquired per tube using either Navios (Beckman Coulter, Brea, CA, USA) or FACS ARIA III (Becton Dickenson (3 lasers–10 colors) flow cytometers. The cytometer was calibrated and monitored daily according to thE manufacture’s procedure and the EuroFlow SOP for instruments [71,72]. For data analysis, the files (.fcs or .lmd) were analyzed using the Infinicyt 1.8 software (Cytognos SL, Salamanca, Spain). The plasmocytes were gated as the population double positive for both CD38 and CD138 markers. A threshold of 10% for all CD was applied to be considered positively expressed on plasmocytes, except for CD19 where the threshold was set at 50% [42].

### 4.3. PCs-Enriching and Genomic DNA (gDNA) Extraction

PCs were purified using the CD138 Plasma Cell Isolation Kit (Miltenyi Biotec GmbH, Bergisch Gladbach, Germany) according to the manufacture protocol [13,75]. Briefly, the whole BM sample was processed for density gradient separation of mononucleated cells (BMMC) with Histopaque^®^-1077 ficoll, (Sigma-Aldrich, St. Loius, MO, USA). After two PBS washes, the BMMCs were resuspended in 80 µL buffer (PBS, pH 7.2, 0.5% BSA and 2 mM EDTA) and incubated with 20 µL CD138 MicroBeads for 15 min. Next, the CD138 cells were washed twice with the same buffer, and finally separated on the magnet using LS columns. After enrichment, the purity of isolated PCs was confirmed to be >80% by flow cytometry using CD38, CD56, CD19 and CD45.

gDNA was extracted with the Wizard^®^ Genomic DNA Purification Kit (Promega Corp., Madison, WI, USA) according to the manufacturer’s instructions [76,77]. The quality and quantity of isolated gDNA was assessed using NanoDrop 2000 (Thermo Fisher Scientific, Waltham, MA,, USA). DNA samples were then stored at −20 °C until use.

### 4.4. MLPA Analysis

All DNA samples were diluted to 20 ng/µL. Then, 5 µL of each diluted DNA sample was subjected to MLPA analysis using SALSA MLPA P425-B1 MM probe mix (MRC-Holland, Amsterdam, The Netherlands) according to the manufacturer’s instructions. The kit contains 46 probes, targeting the following regions: 1p32.3 (FAF1, CDKN2C), 1p32.2 (PLPP3 and DAB1), 1p31.3 (LEPR), 1P31.2 (RPE65), 1p21.3 (DPYD), 1p21.1 (COL11A1), 1p12 (FAM46C), 1q21.3 (CKS1B), 1q23.3 (NUF2, RP11 and PBX1), 5q31.3 (PCDHA1, PCDHAC1, PCDHB2, PCDHB10, SLC25A2, and PCDHGA11), 9p24.1 (JAK2), 9q34.3 (COL5A1), 12p13.31 (CD27, VAMP1, NCAPD2, CHD4), 13q14.2 (RB1 and DLEU2), 13q22.1 (DIS3), 14q32.32 (TRAF3), 15q12 (GABRB3), 15q26.3 (IGF1R), 16q12.1 (CYLD), 16q23.1 (WWOX) and 17p13.1 (TP53). The DNA and the kit probes were denatured for 5 min at 98 °C and hybridized overnight (16–20 h) in a thermocycler, SureCycler 8800 (Agilent Technologies, Palo Alto, CA, USA), followed by ligation (15 min at 54 °C) and PCR (using FAM fluorescence primer). The MLPA products were analyzed using ABI 3500 Genetic analyzer (Applied Biosystems, Foster City, CA, USA) using the default program for peak visualization and Coffalyser software v.140701 (MRC Holland, Amsterdam, The Netherlands) for peak ratio calculation and report generation. In the analysis, for every six samples, two DNA control samples extracted from normal individuals were used. The presence of hyperdiploidy (HRD) was considered if at least one of the odd-numbered chromosomes regions were duplicated [78].

### 4.5. Statistical Analysis

Statistical analyses were performed using the IBM^®^ SPSS Statistics 21.0 Software, R v4.0.3 and Office, Excel software. All parameters were analyzed in relation to progression free survival (PFS) and overall survival (OS). PFS was defined as duration from the start of treatment to disease progression or patient death (regardless of the cause of death). PFS analysis events included disease progression, relapse and patients’ death. Disease progression was diagnosed according to the diagnostic criteria of the IMWG. OS was defined as the time from the date of diagnosis to the date of the patient’s death. OS analysis events included only death. The loss of patients during follow-up was treated as censored information. The Kaplan and Meier method and log-rank test were used for survival analysis. Each figure contains the relevant statistical information: the n, total number of patients and the significance *p*-value; a *p* < 0.05 was considered as statistically significant. The Cox regression analysis was used for generation of the hazard ratio (HR). The elaboration of heat maps was made in R software using the Complex Heatmap package as reported before [79].

## Figures and Tables

**Table 1:** The characteristics of the study group.

The Study Group	Number of Patients (%)
**All patients**	389
MGUS	45 (11.5%)
SMM	46 (11.8%)
Solitary plasmacytoma	13 (3.3%)
Plasma cell leukemia	27 (6.94%)
Other associated diagnoses (e.g., POEMS syndrome or systemic amyloidosis)	102 (26.22%)
Multiple myeloma (MM)	156 (40.1%)
**Characteristics of MM patients investigated by both MFC and MLPA**	**107**
**Age (Years)**	
range 35–69, median 65	
<60	29 (27.1%)
60–70	44 (41.1%)
>70	34 (31.0%)
**Sex**	
Male	51 (47.7%)
Female	56 (52.3%)
**ISS Stage**	
1	21 (19.6%)
2	39 (36.4%)
3	47 (43.9%)
**CRAB informations**	
Anemia	82 (76.6%)
Bone lesions	98 (91.5%)
Hypercalcemia (≥11 mg/dL)	30 (28.0%)
Renal lesions	53 (49.5%)
**Treatment**	
VCD	74 (69.1%)
Other therapies	33 (30.8%)
Autologous transplant	30 (40.5%)

**Table 2:** Copy number alteration (CNA) frequency in the study group.

CNAs	Percent (%)	No. of Samples
HRD	49.5	53
dup1q	40.1	43
del13q	38.3	41
del1p	21.4	23
del16q	19.6	21
del14p	7.5	8
del17p	4.6	5
del12p	1.9	2
none	27.1	29

**Table 3:** Correlation of CD81 expression with adverse CNAs and clinical prognosis of MM patients estimated by Kaplan–Meier and COX regression analysis.

CD81+ Atypical PCs							
Adverse CNAs	*N*	Overall Survival			Progression-Free Survival		
		Months	Hazard Ratio (95% CI)	*p* Value	Months	Hazard Ratio (95%CI)	*p* Value
del13q	23	38	0.39 (0.18–0.85)	0.013	19	0.62 (0.33–1.1)	ns
dup1q	29	38	0.31 (0.14–0.68)	0.002	14	0.38 (0.21–0.7)	0.001
del1p	14	34	0.41 (0.17–0.99)	0.04	16	0.47 (0.23–0.97)	0.035
>3 CNAs	13	38	0.36 (0.15–0.87)	0.017	13	0.46 (0.21–1.0)	0.045

Abbreviations: *N*—number of cases; ns—no statistical significance; *p* < 0.05 was considered statistically significant.

**Table 4:** Pairwise association between maturation stages of PCs and CNAs.

PCs Maturation Stages					Genomic Alterations (CNAs)				
	*N*	del1p	dup1q	HRD	del12p	del13q	del14q	del16q	del17p
CD19 (+) CD81 (+)	4	0%	25%	75%	0%	50%	0%	25%	0%
CD19 (−) CD81 (+)	62	22.6%	43.6%	46.8%	3.2%	33.9%	8.1%	22.6%	6.5%
CD19 (−) CD81 (−)	39	20.5%	33.3%	48.7%	0%	41%	7.7%	12.8%	0%

Abbreviations: *N*—number of cases.

**Table 5:** Correlation of CD19, CD81 and CD117 expression with adverse CNAs and clinical prognosis of MM patients estimated by Kaplan–Meier and COX regression analysis.

CD19, CD81 (PC Maturation Stage)	CNAs	CD117		N	Progression-Free Survival		
		Positive or Negative	Subgroups		Months	Hazard Ratio (Subgroups Compared)	*p* Value (For Subgroups Compared)
CD19− CD81− (differentiated)	<3	+	(a)	22	>48	1.88 (0.22–15.69)	-
		−	(b)	10	24	0.68 (0.06–7.70) (b vs. a)	0.75
	≥3	+	(c)	5	>48	0.56 (0.06–4.48) (c vs. a)	0.53
		−	(d)	2	13	x	x
CD19− CD81+ (intermediate)	<3	+	(e)	27	>48	3.021 (1.28–7.08)	-
		−	(f)	23	19	0.33 (0.14–0.77) (f vs. e)	0.008
	≥3	+	(g)	9	13	0.35 (0.12–1.04) (g vs. e)	0.049
		−	(h)	3	7	0.21 (0.042–1.05) (h vs. e)	0.051
CD19+ CD81+ (little or non-differentiated)	<3	+	(i)	3	17	0.28 (0.76–1.09) (i vs. e)	0.052
	≥3	−	(j)	1	7	x	x

Note: For each subgroup of PCs, a letter (a to j) was assigned in order to easily follow the comparations between subgroups. x—for these groups no statistical was performed due to the low number of patients.

## Data Availability

Supplementary data are available on request at shorturl.at/eHIMO, lucian.gorgan@uaic.ro.

## Appendix B

**Table A1:** CD marker frequency.

Makers	Percentages	Number of Samples Investigated
CD81+	62.8% (*n* = 66)	105
CD45+	85.04% (*n* = 91)	107
CD56+	82.2% (*n* = 88)	107
cyIgK	63.55% (*n* = 68)	107
cyIgL	36.44% (*n* = 39)	107
CD28+	60.7% (*n* = 62)	102
CD27+	63% (*n* = 63)	100
CD117+	63.2% (*n* = 67)	106
